# Science that speaks: The public face of physiology

**DOI:** 10.1113/EP093219

**Published:** 2025-10-01

**Authors:** Danny W. Walmsley, Daniella Hurt, Abdulrahman A. Dahesh, Kate Williams, Damian M. Bailey

**Affiliations:** ^1^ Neurovascular Research Laboratory, Faculty of Life Sciences and Education University of South Wales Pontypridd UK; ^2^ Department of Communications, Public Affairs and Policy, Faculty of Life Sciences and Education University of South Wales Pontypridd UK; ^3^ Bexorg Inc. New Haven Connecticut USA

## 1 INTRODUCTION

**FIGURE 1:**
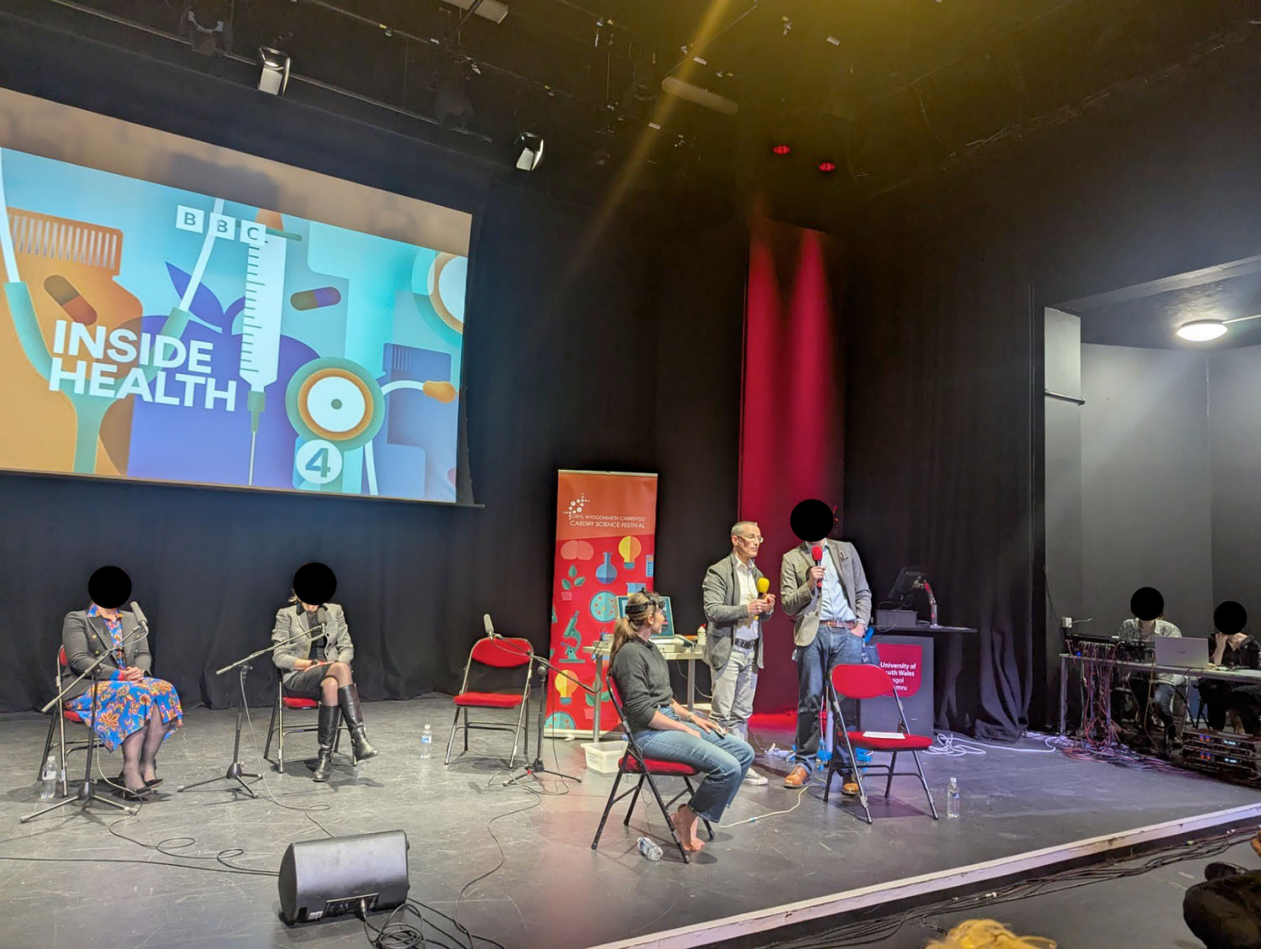
BBC Inside Health: Will wearables revolutionise healthcare? Cardiff Science Festival Special. *Photo courtesy of Kate Williams*.

In an era marked by escalating social media usage and the rapid spread of misinformation (Carrieri et al., 2023; Dadaczynski et al., 2021; Vosoughi et al., 2018), the need for physiologists to directly disseminate robust, research‐backed insights from accessible and transparent research (Berg et al., 2024; Rasmussen et al., 2025) on key health issues has become increasingly important. Our recent participation in a live BBC Radio 4 segment for Inside Health (www.bbc.co.uk/sounds/play/m0028jkm and Figure 1), centred on wearables for health data collection, vividly demonstrated how direct engagement can transcend traditional learning barriers and foster understanding between physiological researchers and the public.

The physiologist–public interface offers significant benefits to both parties: researchers gain the opportunity to share novel physiological insights, potentially attracting funding and fostering new collaborations, while the public benefits from a deeper understanding of relevant physiological concepts, enabling more informed decisions to improve their health and well‐being. In the current editorial, we take the opportunity to shine a light on some of the key benefits and challenges currently facing our physiological community.

## 2 BENEFITS TO THE PHYSIOLOGIST

Researchers who engage more frequently with society perform better academically, challenging the ‘Sagan effect’ – the notion that public‐facing academics produce research of lesser quantity or quality. However, a comprehensive analysis of researchers at the French National Centre for Scientific Research identified a positive relationship between public engagement and academic performance (Jensen et al., 2008). Despite this, there are reports of particularly junior researchers being discouraged from sharing expertise through popular articles in non‐academic outlets (Martinez‐Conde, 2016). This discouragement often stems from current academic incentive systems that primarily reward traditional outputs, notably peer‐reviewed journal publications, and do not yet formally recognise or adequately reward the time and effort that researchers invest in public communication exploits.

Engagement with mainstream media can also help to raise a researcher's profile not only within his/her own discipline, but across domains and often outside of physiology itself. This can manifest as unforeseen collaborations with, for example, bioinformaticians to analyse population data, invitations to advise on public health policy, or industry partnerships to translate a discovery from the laboratory to clinical application. Equally, engagement with the public necessitates not only developing communication skills but also deepening one's subject knowledge in preparation for those inevitable and often difficult‐to‐answer questions. There is also potential for a ‘protégé effect’ by which those who communicate and educate on a given subject enhance their own understanding and memory (Chase et al., 2009; Nestojko et al., 2014).

## 3 BENEFITS TO THE PUBLIC

Physiologists, through media exposure, can arm the public with research‐backed information and teach lay people how to identify high‐quality information sources amidst a deluge of online misinformation (Berg et al., 2024). This leads to more informed choices around diet, exercise and lifestyle (Nutbeam, 2000). Our recent BBC Radio 4 ‘Inside Health’ segment (Figure 1), for example, engaged the audience in a live physiology practical, guiding them through a controlled breathing exercise while they tracked their own heart rates on wearable devices. We then performed a cold pressor test, with a consenting laboratory member as the participant, whom we chose to be female, given the current under‐representation (Bailey, 2023), to demonstrate dynamic, integrated changes in anterior/posterior cerebral blood flow, pulmonary ventilation, heart rate and arterial blood pressure (Flück et al., 2017). Putting sounds to the numbers was especially impactful for the audience!

Such live demonstrations provide uniquely memorable insight into physiological mechanisms for non‐experts, making abstract concepts immediately tangible and engaging audience participation. Indeed, an informed public is better equipped to engage with public policies on issues of healthcare, scientific policy, funding priorities and the ethics of emerging issues and potential solutions. This converts citizens from passive recipients of healthcare and physiological research into active participants in shaping their future.

## 4 CHALLENGES

However, a core challenge in the communication of physiology is the translation of complex terminology and mechanisms into layman's terms. In their public engagements, physiologists are advised to avoid ‘talking shop’ and using field‐specific names, jargon and abbreviations in favour of more palatable language (Brownell et al., 2013). This may entail strategies such as storytelling, relevant analogies, and effective visual diagrams to communicate information in accessible and engaging formats. Effective translation requires a genuine effort to understand the audience's existing knowledge, perspectives, concerns and potential misconceptions, such as surveying the audience's current use of wearable technologies in the Inside Health segment.

Among these strategies, storytelling warrants special attention for its unique ability to weave complex scientific information into emotionally engaging and memorable forms. As Dahlstrom (2014) notes, narratives differ from traditional expository communication by embedding facts within a structure that taps into emotions, values and prior experiences, thereby increasing both comprehension and recall.

The effectiveness of storytelling can be explained, in part, by the transportation process (Green & Brock, 2000) in which audiences become absorbed in a narrative world, lowering counter‐arguing and increasing openness to new ideas. This persuasive potential has been demonstrated in contexts such as shaping public beliefs on vaccines (Brodie et al., 2001) and HIV/AIDS (Dahlstrom, 2014).

Effective science storytelling is the strategic translation of complex science into relatable experiences. Frameworks like the SUCCESS principles (Simple, Unexpected, Concrete, Credible, Emotional, Science Storytelling; Finkler & León, 2019) offer practical guidance for crafting narratives that could change people's attitudes and intentions. This could mean narrating a real case study to illustrate how a physiological principle manifests clinically or using fictional scenarios (especially for younger audiences) to explore complex concepts, including exercise‐induced changes in substrate delivery to the brain and its translational links to altered structure and (cognitive) function.

However, this power demands responsibility. While stories enhance engagement with science (Dahlstrom, 2014), they inherently risk oversimplification or introducing unintended misconceptions if scientific rigour is sacrificed for narrative flow (Dahlstrom & Ho, 2012). Physiologists must move beyond solo efforts towards deliberate narrative design, potentially collaborating with skilled science communicators or instructional designers.

Physiologists also need to contend with an overwhelming volume of misinformation, particularly on social media platforms. One psychological phenomenon fuelling the spread of false narratives is the ‘illusory truth effect’, by which the repeated exposure to a statement, regardless of its validity, increases its perceived truthfulness (Hasher et al., 1977). To effectively counter this, physiological communication needs to employ strategies that ensure accurate information is both impactful and memorable. This involves not only strategic debunking – explaining the rhetorical techniques used by misinformation campaigners rather than just reiterating facts (Lewandowsky et al., 2012) – but also ‘pre‐bunking’, a form of inoculation against misinformation techniques, in part through educating the public on methods of identifying reliable information sources (Drummond & Tipton, 2024; van der Linden et al., 2017).

Engaging with the public is not without its risks to the researcher, however. Researchers who engage with the public, particularly through online platforms, are at significant risk of harassment, disagreement and ‘trolling’ (the act of commenting on a person or subject on the internet, solely to evoke upset) from the public. A recent popular example is that of the University of Cambridge's Dr Ally Louks, who, after posting an image holding her completed doctoral thesis entitled ‘Olfactory Ethics: The Politics of Smell in Modern and Contemporary Prose’, was subjected to a wave of online harassment and threats.

Our recent article published in The Conversation, which was intended to raise awareness of the cerebrovascular consequences of a high‐fat meal (the aptly‐named ‘Bailey‐Brain‐Bomb’ milkshake) (https://theconversation.com/we‐fed‐people‐a‐milkshake‐with‐130 g‐of‐fat‐to‐see‐what‐it‐did‐to‐their‐brains‐heres‐what‐we‐learned‐259961), and served as a follow‐up to our published study (https://www.sciencedirect.com/science/article/pii/S3050624725000051), was also met with public criticism from readers who objected to being cautioned against high‐fat fast foods. This harassment has both apparent first‐hand and less obvious second‐hand effects on the public's perception of science. As highlighted by Egelhofer et al. (2024), the increased volume and visibility of disagreement with, and harassment of, researchers may also negatively impact the public's perception of scientists.

## 5 NEXT STEPS

The lack of formal recognition of researchers who choose to disseminate their knowledge through public channels could harm both the researcher and wider society. This shift involves cultivating institutional cultures where public communication is viewed as an integral alter‐dimension of scholarly activity. This issue was recently discussed by Mohammad et al. (2025), who highlighted the limitations of relying solely on citation‐based metrics such as the H‐index to assess genuine societal impact. Part of this involves equipping the messenger – physiologists with the necessary skills to effectively engage with the public and media. Comprehensive media and communication training should be integrated at all career levels, from graduate studies to senior faculty development, with a focus on translational impact, one of our key research metrics.

Finally, effective collaborations between physiologists, institutions and media organisations need to be actively fostered. This involves moving beyond simple transactional interactions, such as a press release for a paper or a brief quote for a news article, towards more relational engagements built on trust, respect and mutual commitment to informing the public accurately and responsibly. This can be attained through the appointment of institutional press officers trained in science communication, clear pathways for journalists to access expert physiologists for timely fact‐checking, and initiatives that promote mutual understanding of each other's professional norms, constraints and objectives. Additionally, this may entail the development of dedicated institutional blogs, podcasts and video series to provide reliable information to the public.

If physiology is to fulfil its potential in improving lives, it must be heard beyond the laboratory. Engaging with the media transforms our science from static knowledge into a dynamic public power – bridging the gap between discovery and impact, and making physiology not just known, but felt. Lights, camera, action, physiological impact!

## AUTHOR CONTRIBUTIONS

*Conceiving the idea and writing the first draft of the manuscript*: Damian M. Bailey and Danny W. Walmsley. *Editing and revising of the manuscript*: Danny W. Walmsley, Daniella Hurt, Abdulrahman A. Dahesh, Kate Williams and Damian M. Bailey. All authors have read and approved the final version of this manuscript and agree to be accountable for all aspects of the work in ensuring that questions related to the accuracy or integrity of any part of the work are appropriately investigated and resolved. All persons designated as authors qualify for authorship, and all those who qualify for authorship are listed.

## CONFLICT OF INTEREST

D.M.B. is Editor‐in‐Chief of *Experimental Physiology*, Chair of the Life Sciences Working Group, member of the Human Spaceflight and Exploration Science Advisory Committee to the European Space Agency, member of the Space Exploration Advisory Committee to the UK and Swedish National Space Agencies and member of the National Cardiovascular Network for Wales and South‐East Wales Vascular Network.

## FUNDING INFORMATION

D.M.B. is supported by a Royal Society Wolfson Research Fellowship (Grant No. WM170007).

## Data Availability

The data that support the findings of this study are available from the corresponding author upon reasonable request.
